# The Impact of the COVID-19 Pandemic on Injury Patterns in Inpatient and Outpatient Orthopaedic Trauma

**DOI:** 10.1055/s-0042-1757427

**Published:** 2022-12-26

**Authors:** Thomas Gatt, Sharon Zammit, Kurt L. Chircop, Denise Gatt, Luke Sultana, Terence Micallef, Adriana Grech, Ivan Esposito

**Affiliations:** 1 Department of Orthopaedics and Trauma, Mater Dei Hospital, L-Imsida, Malta

**Keywords:** COVID-19, trauma, injury patterns, outpatients, injury mechanics

## Abstract

The effect of banning of nonessential services during the novel coronavirus disease 2019 (COVID-19) pandemic led to a perceived change in the volume of trauma cases and injury patterns. Literature indicates trends toward less trauma overall, with a decrease in outdoor, sporting, and motor vehicle injuries. However, studies focusing on outpatient orthopaedic trauma are less common. The main aim of this study was to assess the effect of COVID-19 pandemic on differences in inpatient and outpatient injury patterns and mechanisms. Patients requiring orthopaedic inpatient admission to Mater Dei Hospital, Malta, were analyzed between March 15 and June 17 between 2019 and 2021. For outpatients, all newly referred patients seen at the fresh trauma clinic (FTC) on the first 5 clinic days of each month from April to June between 2019 and 2021 were assessed. There were a total of 503, 362, and 603 hospital admissions during the data collection period from 2019, 2020 and 2021, respectively. There was a decrease in elbow ( *p* = 0.015) and pelvis ( *p* = 0.038) pathology since COVID-19 pandemic. In contrast, there was an increase in shoulder injuries ( *p* = 0.036) and lacerated wounds ( *p* = 0.012) in 2021. The most frequent mechanisms of injury for inpatients were low impact falls, and fall from heights greater than 1 m. Machine-related injuries ( *p* = 0.002), blunt trauma ( *p* = 0.004), and twisting injuries ( *p* = 0.029) increased in 2021. In the outpatient setting, there were a total of 367, 232, and 299 new referrals in 2019, 2020, and 2021, respectively. Injury patterns in this cohort were similar throughout, except for a significant increase in shoulder injuries during 2020 ( *p* = 0.009). There appears to be some minor variation in injury mechanisms due to lifestyle changes; however, most injury patterns have remained fairly constant. Further research should focus on the use of public awareness campaigns to decrease home-related trauma during enforced periods of lockdown.

The novel coronavirus disease 2019 (COVID-19) pandemic brought around unprecedented strain and challenges on health care systems around the world. Like most other countries, Malta saw widespread cancellations of elective surgical work, with priority toward emergency life or limb saving surgery. This was done in an attempt to free up hospital capacity for the influx of COVID-19 patients. 1 Furthermore, in an effort to increase the turnover of urgent surgical cases and minimize time in hospital, the frequency of daily general emergency and orthopaedic trauma theaters were doubled. The orthopaedic trauma care in Malta saw its biggest impact with the government's introduction of national lockdown on March 17, 2020. 2 With the banning of nonessential services, such as restaurants and shops, this saw more people staying off the streets and more time indoors. This led to a perceived change in the number of cases presenting to the Emergency Department, as well as the types of injury patterns seen.

This topic has been the subject of research by numerous institutions across the world. 3 4 5 6 7 For instance, a reduction in orthopaedic trauma ranging from 20 to 85% was reported in a systemic review by Waseem et al which looked at trauma patterns from across Europe, Asia, the United States, New Zealand, and Australia. 8 Similarly, Lim et al reported a decrease in trauma of around 30 to 50% in their meta-analysis, while acknowledging a higher injury severity and an increased mortality rate in fractures. 3 7 The trends described seem to be toward less outdoor and sporting injuries, as well as decreased traffic accidents. 8 One study found the reduction in motor vehicle accidents (MVAs) to be as high as 40%. 4 6 Contrarily, increased incidence of slip and fall injuries, domestic work injuries, domestic violence and self-harm were observed. 3 6 8 Specifically, one study reported higher incidence in hip and spine fractures. 6

While the incidence of minor injuries seeking help decreases in these situations, the subsequent proportion of moderate and major trauma increased which justifies the emphasis placed on maintaining efficient trauma care during a pandemic. 5 9 Since the frequency of pandemics are expected to increase in the future, analyzing the local trends of orthopaedic trauma in these situations is beneficial to plan resource management. 10 It can also serve as a reference point on where to direct orthopaedic training efforts in the future. Thus, the primary aim of this study was to assess the differences in injury patterns and trauma mechanisms brought about by the COVID-19 pandemic for both inpatients and outpatients, compared with the overall observed yearly variation. The secondary aims were to assess the effect of running two simultaneous trauma theaters on the time to definitive surgery and the length of hospital stay.

## Methods

For hospital admissions, all patients with acute orthopaedic trauma requiring inpatient admission to Mater Dei Hospital, Malta, were analyzed. The time period chosen was from March 17 to June 17 for 3 consecutive years between 2019 and 2021. This was chosen since March 17, 2020 coincided with the government-enforced nationwide lockdown which had been lifted by the following year. For outpatients, all newly referred patients seen at the fresh trauma clinic (FTC) on the first 5 working days of each month from April to June, for 3 consecutive years between 2019 and 2021 were assessed. This was chosen to coincide with trauma sustained approximately 2 weeks prior and mirror the inpatient cohort analysis date.

The data was collected retrospectively and prospectively from several sources including the Malta Orthopaedic Trauma Registry, Centricity Universal Viewer Zero Footprint software and iSoft Clinical Manager (iCM) software. Data parameters include patient demographics, date of presentation, type of injury sustained, mechanism of injury, time to surgery or first review at FTC and patient disposition. SPSS software was used to analyze data. Pearson's Chi-squared test for 3 × 2 contingency tables were used to assess for difference between the observed and expected dependent variables (injury mechanism) across the categorical variables. Post hoc comparisons were also performed to assess for difference between two independent categorical variables. Analysis of variance (ANOVA) testing was used for quantitative variables. Moods median test was used to assess median length of stay (LOS). A *p* -value of <0.05 was deemed statistically significant. Ethics approval was obtained from the Faculty of Medicine and Surgery Research Ethics Committee.

## Results

There were a total of 503, 362, and 603 hospital admissions during the data collection period between 2019, 2020, and 2021, respectively. The admission rate decreased by 28.0% between 2019 and 2020, and subsequently increased by 66.6% between 2020 and 2021. There were no significant differences between the mean age ( *p* = 0.239), male-to-female ratio ( *p* = 0.881), median LOS ( *p* = 0.054) or inpatient mortality ( *p* = 0.534) between the three groups ( Table 1 ). There was significant decrease in the conservative treatment rate from 2020 onwards ( *p* < 0.001). Mean time to surgery in 2020 was 2.36 ± 0.27 days, and while this was less when compared with 2019 or 2021, this was not statistically significant ( *p* = 0.079)

**Table 1:** Patient demographics and inpatient quality indicators

	2019 Mean ± SD/ *n* (%)	2020 Mean ± SD/ *n* (%)	2021 Mean ± SD/ *n* (%)	*p* -Value
Admissions ( *n* )	503	362	603	
Mean age (y)	54.02 ± 2.15	56.6 ± 2.49	54.34 ± 1.98	0.239
M/F ratio	1.38	1.27	1.32	0.881
Median LOS (d)	5 (8)	4 (9)	5 (9)	0.054
Conservative treatment	108 (21.5)	45 (12.4)	87 (14.4)	<0.001
Mean time to surgery (d)	2.85 ± 0.38	2.36 ± 0.27	2.9 ± 0.34	0.079
Inpatient mortality	7 (1.39)	5 (1.38)	13 (2.15)	0.534

Abbreviations: F, female; LOS, length of stay; M, male; SD, standard deviation.

The most prevalent injury patterns were hip, wrist, and ankle pathology. There was a decrease in elbow ( *p* = 0.015) and pelvis ( *p* = 0.038) pathology since COVID-19 when compared with 2019. In contrast there was an increase in shoulder injuries ( *p* = 0.036) and lacerated wounds (LCWs; *p* = 0.012) in 2021 when compared with the annual incidence variation ( Table 2 ). The most frequent mechanisms of injury for in-hospital patients were low impact falls from own heights, and fall from heights more than 1 m. There was a statistically significant decrease in sporting injuries during 2020 and 2021 when compared with 2019 ( *p* = 0.002). Machine-related injuries ( *p* = 0.002), blunt trauma ( *p* = 0.004), and twisting injuries ( *p* = 0.029) were higher in 2021 when compared with previous years ( Table 3 ).

**Table 2:** Injury patterns for in-hospital patients

Injury patterns	2019 ( *n* )	2020, *n* (% change in incidence from preceding year)	2021, *n* (% change in incidence from preceding year)	*p* -Value
Hand	35	23 (−8.7)	39 (+1.8)	0.936
Wrist	74	49 (−7.99)	67 (−17.9)	0.181
Elbow	37	14 (−47.4)	23 (−1.4)	0.015 ^*^
Shoulder	21	16 (+5.9)	45 (+68.8)	0.036 ^*^
Spine	25	11 (−38.8)	25 (+36.4)	0.400
Pelvis	22	6 (−62.1)	14 (+40.0)	0.038 ^*^
Knee a	44	39 (+23.2)	54 (−16.9)	0.488
Hip	121	102 (± 17.1)	146 (−14.1)	0.228
Ankle	53	45 (± 18.0)	80 (+6.7)	0.372
Infections b	39	20 (−28.7)	33 (−0.9)	0.248
Abscesses	10	10 (+38.9)	21 (+26.1)	0.327
LCWs	24	23 (+33.2)	56 (+46.1)	0.012 ^*^
Miscellaneous	23	15 (−9.4)	31 (+24.1)	0.799

Abbreviation: LCW, lacerated wound.

a Knee: including distal femur/proximal tib* proximal tibia.

b Infections; septic joints, cellulitis.

*Statistically significant difference between the three groups noted.

**Table 3:** Injury mechanisms for in-hospital patients

Injury patterns	2019 ( *n* )	2020 *n* (%)	2021 *n* (%)	*p* -Value
FFH	71	39 (−23.7)	68 (+4.7)	0.234
FOH	188	148 (+9.4)	205 (−16.9)	0.095
FOOSH	35	26 (+3.2)	40 (−7.6)	0.944
Blunt trauma	7	4 (−20.6)	24 (+260)	0.004 ^*^
Sports injuries	16	4 (−65.3)	7 (+5.1)	0.022 ^*^
Machine related	10	12 (+66.7)	37 (+85.1)	0.002 ^*^
MVA	38	34 (+24.3)	61 (+7.7)	0.325
Postoperative	9	2 (−69.1)	8 (+140.1)	0.282
Crush injury	25	14 (−22.2)	20 (−14.2)	0.373
Penetrating injury	22	13 (−17.9)	18 (−16.9)	0.467
Twisting injury	9	5 (−22.8)	23 (+176.2)	0.029 ^*^
Foreign body	3	2 (−7.4)	5 (+50.1)	0.844
Assault	5	4 (+11.2)	5 (−25.0)	0.739
Spontaneous	20	12 (−16.6)	35 (+75.1)	0.147
Infective	10	10 (+38.6)	14 (−16.0)	0.757
Miscellaneous	11	15 (+89.5)	15 (−40.0)	0.190
Unknown	24	18 (+4.2)	19 (−36.6)	0.270

Abbreviations: FFH, fall from height, any vertical drop more than 1 m; FOH, fall own height: any mechanical fall less than 1 m; FOOSH, fall on outstretched hand; MVA, motor vehicle accident.

*Statistically significant difference between the three groups noted.

There were a total of 367, 232, and 299 new fracture clinic referrals in 2019, 2020, and 2021, respectively. The referrals decreased by 36.8% between 2019 and 2020, while they increased by 28.9% between 2020 and 2021. The mean age was statistically different between the three groups, with an older mean age in the patient seen since the start of the pandemic. ( *p* = 0.007). Median time to review in 2020 was 9.0 days (interquartile range [IQR]: 8.0–11.0 days) which was significantly lower compared with 2019 and 2021. ( *p* < 0.001). Injury patterns in this patient cohort were similar in comparison to annual incidence variation, except for a significantly higher rate of shoulder injury patterns during 2020 ( *p* = 0.009; Table 4 ). There were a lower proportion of sports injuries ( *p* = 0.030), twisting injuries ( *p* = 0.020), and crush injuries ( *p* = 0.010) during 2020. Low impact mechanical falls from height were significantly higher since the start of the pandemic ( *p* < 0.001). There was no difference between MVAs occurring in the past three years. ( *p* = 0.108; Table 5 ).

**Table 4:** Injury patterns for outpatient cohort

Injury patterns	2019 *n* (%)	2020 *n* (%)	2021 *n* (%)	*p* -Value
Hand	49 (15.4)	36 (+16.2)	47 (+1.3)	0.596
Wrist	68 (22.7)	40 (−7.0)	52 (+0.9)	0.833
Elbow	26 (7.6)	13 (−20.9)	24 (+43.3)	0.587
Shoulder	47 (14.7)	50 (+68.3)	44 (−31.7)	0.009
Spine	7 (1.9)	6 (+35.6)	11 (+42.3)	0.153
Pelvis	6 (1.7)	2 (−20.9)	3 (+16.4)	0.961
Knee	10 (2.8)	2 (−68.4)	9 (+249)	0.230
Ankle	42 (12.9)	14 (−47.3)	23 (+27.5)	0.062
Foot	36 (10.9)	32 (+40.6)	34 (−17.6)	0.270
Other	19 (5.5)	8 (−33.4)	11 (+6.7)	0.523
Unknown/no fracture	57 (18.4)	24 (−33.4)	40 (+29.3)	0.229

**Table 5:** Injury mechanisms for outpatient cohort

Injury mechanisms	2019 ( *n* )	2020 *n* (%)	2021 *n* (%)	*p* -Value
FFH	12	13 (+71.4)	18 (+7.4)	0.192
FOH ^*^	59	70 (+87.7)	70 (−22.4)	<0.001 ^*^
FOOSH	71	40 (−10.9)	58 (+12.5)	0.840
Blunt trauma	89	52 (−7.6)	56 (−16.4)	0.227
Crush injury	24	11 (−27.5)	5 (−64.7)	0.010 ^*^
Sports injuries	27	6 (−64.8)	13 (+68.1)	0.030 ^*^
Twisting injuries	45	12 (−57.8)	31 (+100.4)	0.020 ^*^
MVA	10	10 (+58.2)	18 (+39.7)	0.108
Other	20	9 (−28.8)	20 (+72.4)	0.392
Unknown	10	4 (−36.7)	9 (+74.6)	0.649

Abbreviations: FFH, fall from height, any vertical drop more than 1 m; FOH, fall own height: any mechanical fall less than 1 m; FOOSH, fall on outstretched hand; MVA, motor vehicle accident.

*Statistically significant difference between the three groups noted.

## Discussion

Mater Dei Hospital is the main acute general hospital in Malta and sees most of all orthopaedic trauma. Similar to other countries at the height of the pandemic, Malta followed suit in introducing several recommended operating procedures. One example was mobilizing orthopaedic staff down to the emergency department to help with the reviewing of patients in an attempt to decrease patient time spent in casualty. 11 Another key aspect was decreasing the volume of patients returning for follow-up by using adjuncts such as absorbable sutures or removable splints where possible. 12 This was augmented by placing a big emphasis on teleconsultations. 1 6 However, these changes, combined with postponing elective work and doubling trauma theater, had little impact on the median LOS and mean time to surgery. It may be argued that there would have actually been a decrease in LOS or time to surgery, had this not been offset by the increased patient turnover time during COVID-19. This may have arisen from the need to wait for COVID-19 swab results and lengthy protocols for operating in a COVID-19 theater.

While there are many studies documenting incidence of orthopaedic trauma during the pandemic, this study is one of the few that also looks at the impact of this simultaneously on an outpatient trauma service. For instance, there was a noted decrease in the conservative treatment rate since the beginning of the pandemic. This was attributed to the increased attempt to discharge people from A&E who do not really require admission to prevent overloading the hospital. While the practice had been to admit borderline cases for surgery and discuss with the caring consultant at the trauma meeting the following day, emphasis during COVID-19 was placed on having a management plan prior to admission, ensuring patients admitted were almost certainly for surgery. While one would expect to see a corresponding increase in outpatient FTC reviews, this was not the case. The probable cause for this is that patients previously suffering minor trauma were not seeking medical advice for fear of contracting COVID-19. In contrast, there was an increase in patients admitted with LCW admissions. Prior to the pandemic, some minor cases may have been managed in a minor theater room in casualty. However, to decrease health worker exposure unnecessarily, anything which was deemed necessary for theater was admitted as opposed to attempting exploration and repair in casualty.

The decrease in sporting injuries coincides with the government's decision to ban all sporting events in keeping with practice around the globe. 13 This study confirms the expected decrease in sporting injuries as a result. Interestingly, despite more people working from home, decreased outdoor activities with a resultant decrease in traffic on the roads, the MVA rates remained relatively constant. 14 The increased incidence of low impact domestic falls was in keeping with more people being confined in doors. Whether or not this was simply related to more time spent indoors as opposed to an increased injury risk per time spent indoors was unable to be assessed. However, this source of trauma could easily be targeted in future events necessitating people to stay at home. It would be beneficial if part of the public health announcements would give occupational therapy advice on how to decrease risk factors for injuries in the household. For instance, elderly patients in the community might be counselled in removing rugs, having safety mats in the bathroom or wearing appropriate footwear. Furthermore, televised physiotherapy exercise at home would be useful in keeping the elderly fit. 15 16

## Strengths and Limitations

This study is novel as it assesses the impact of COVID-19 on injury mechanism and pattern, distinguishing between an inpatient and outpatient cohort of orthopaedic trauma. The fact that our institution caters for almost the entirety of trauma locally, implies that our findings are representative of the whole population. The main limitations of this study lie in its retrospective design. There were several record keeping issues, and poor documentation of clinical details in requests for medical imaging, especially in the outpatient cohort. This at times led to difficulty ascertaining the exact mechanisms of injury. There is also concern that as multiple injury patterns and mechanisms are being investigated, this may give rise to type I errors. Indeed lowering the significance level to a more conservative *α* value of 0.01 would negate some significant findings relating to injury mechanisms. The 3-monthly data collection periods were chosen to assess the immediate impact of government lockdown. This may, however, introduce an element of bias by excluding seasonal variation in trauma. Expanding the data collection to an entire 3-year period was technically unfeasible, given the patient volume.

## Conclusion

Like many other centers abroad, Malta saw a decrease in orthopaedic trauma during 2020. This has since recovered and superseded pre-COVID-19 figures. There appears to be some minor variation in injury mechanisms due to lifestyle changes, such as increased sporting events and more low impact falls from own height at home. Despite this, most of the injury patterns have remained fairly constant. Further research should investigate the use of public awareness campaigns to decrease home-related trauma during enforced periods of lockdown.
